# Acupuncture combined with traditional Chinese medicine e-aid cognitive behavioral therapy for insomnia (TCM-eCBT-I) for chronic insomnia: study protocol for a randomized controlled trial

**DOI:** 10.1186/s13063-022-06012-6

**Published:** 2022-01-28

**Authors:** Cheng-yong Liu, Ya-nan Zhao, Xiao-qiu Wang, Shan Qin, Qing-yun Wan, Shi-yu Zheng, Wen-zhong Wu

**Affiliations:** 1 Jiangsu Province Hospital of Chinese Medicine, Affiliated Hospital of Nanjing University of Chinese Medicine, No. 155, Hanzhong Road, Qinhuai District, Nanjing, Jiangsu China; 2 Institute of Acupuncture and Moxibustion, China Academy of Chinese Medical Sciences, Beijing, China

**Keywords:** Acupuncture, e-aid cognitive behavioral therapy for insomnia, Chronic insomnia, PSQI, Randomized controlled trial

## Abstract

**Introduction:**

The incidence of insomnia is getting higher and higher. Long-term insomnia seriously affects people’s health. Drug use is usually accompanied with adverse events. Both acupuncture and cognitive behavioral therapy for insomnia (CBT-I) have been proven to be safe and effective non-pharmacological treatments for insomnia. As the insomniacs’ bad sleep behavior and wrong cognition have not been effectively corrected, acupuncture has a quick effect, high patient compliance but unstable long-term efficacy, while CBT-I is complex, time-consuming, and expensive; additionally, patient compliance is low, and the number of trained therapists is limited, making it difficult to carry out. Therefore, this study aims to use the insomnia TCM system to construct a convenient and feasible traditional Chinese medicine e-aid cognitive behavioral therapy for insomnia (TCM-eCBT-I) for Chinese people, and combine the advantages of acupuncture and TCM-eCBT-I for maintaining long-term efficacy, and three treatments will be evaluated to provide clinicians with a more effective clinical protocol

**Methods and analysis:**

This study is a single-center, open-label, randomized controlled trial. Ninety subjects will be recruited and randomly assigned to three groups: the acupuncture group, the TCM-eCBT-I group, and the acupuncture combined with TCM-eCBT-I group, in a ratio of 1:1:1. We will evaluate the Pittsburgh Sleep Quality Index (PSQI) and Dysfunctional Beliefs and Attitudes About Sleep Scale (DBAS), Insomnia Severity Index (ISI), sleep diary, Hamilton Anxiety Scale (HAMA), Hamilton Depression Scale (HAMD), and Fatigue Scale-14 Scale (FS-14) scales. All adverse reactions will be assessed through the ADVERSE event table. All outcomes will be evaluated online at 0 weeks, 4 weeks, 8 weeks, 16 weeks, and 28weeks.

**Ethics and dissemination:**

This study has been approved by the Institutional Review Board of the Affiliated Hospital of Nanjing University of Chinese Medicine (2020 NL-018-02). Informed consent will be obtained from all the subjects. The results will be shared with sleep researchers, public, and relevant academic institutions through high-impact peer-reviewed publications.

**Trial registration:**

Chinese Clinical Trial Registry ChiCTR2000032960. Registered on 17 May 2020

## Strengths and limitations

- A single-center, open-label, randomized controlled trial strictly follows the principles of CONSORT and STRICTA.
- It is the first time to investigate the long-term efficacy and safety of acupuncture combined with TCM-eCBT-I in treating chronic insomnia.
- Subjects will be conveniently evaluated online and followed up for a long time through the insomnia TCM system.
- Recruitment of this study is limited to one center of Chinese first-class hospitals, and the sample size is too small so that the results may not be applicable to other levels of hospitals or other countries.
- This study cannot blind subjects, which may lead to bias and affect the results.

## Background

With fierce social competition, accelerated pace of life, and the increasing mental stress, the incidence of insomnia is getting higher and higher. About 10 to 20% of people in the world suffer from insomnia, while the prevalence of insomniacs in adult is 33 to 50% [1, 2]. Long-term insomnia affects the neuroendocrine immune network system, resulting in multiple organ dysfunction and decreasing immune function and is often accompanied by anxiety and depression, daytime fatigue, cognitive dysfunction, and other injuries [3–5]. It can even cause traffic accidents, endanger personal and public safety, and place heavy burden on society and families [6–8]. Therefore, it is crucial for both individuals and the public to keep a healthy sleep without insomnia.

At present, there are many treatments for insomnia. Drug therapy is convenient, fast-acting, and still the most common treatment for insomnia [9]. However, a large number of studies have shown that long-term drug use is usually accompanied with adverse events, including ineffectiveness, drug resistance, forgetfulness, nightmare, and cognitive impairment [10]. Most insomniacs are eager to seek effective and safe treatments for insomnia. In the past two decades, non-pharmacological treatments have attracted increasing attention [11].

Acupuncture, as a natural therapy with a long history, has been widely used in Asian countries such as China and South Korea, because of its advantages of safety, effectiveness, and convenience [2]. According to a survey of private clinics, insomnia is one of the top ten acupuncture indications in the USA [12]. In recent years, more and more evidence has shown that acupuncture is safe and effective in treating insomnia [13–19]. Acupuncture is also included in Chinese guidelines for the diagnosis and treatment of insomnia [20]. However, as patients’ bad sleep behavior and wrong sleep cognition are not effectively corrected, the effect of acupuncture is usually short-term. In addition, most clinical studies seldom include long-term follow-up. Thus, the long-term effect of acupuncture on insomnia is still unclear.

Most insomnia diagnosis and treatment guidelines recommend CBT-I as the first choice [20–22]. Some meta-analyses suggest that CBT-I is effective for primary insomnia [23] and comorbidity insomnia [24]. It can improve sleep quality by changing the wrong cognition and behavior factors of insomniacs and can enhance confidence in self-controlled insomnia [25]. Compared with drugs, CBT-I can improve sleep quality for up to 3 years, with minimal risks [23, 26]. Up to now, traditional face-to-face CBT-I usually needs 8 interviews, which is time-consuming, laborious, and expensive. In addition, the number of therapists who have received formal training is limited. In fact, only a few patients can benefit from CBT-I [27]. Both acupuncture and CBT-I have proven to be safe and effective non-pharmacological treatments for insomnia. Most acupuncture studies usually choose positive drugs or placebo as control. Only a few randomized controlled trials directly compare acupuncture and CBT-I in treating insomnia, but the conclusion is still unclear [28]. With the rapid development of the Internet, in order to improve the feasibility of CBT-I and to benefit more patients, eCBT-I through personal intelligent terminals has become a trend in insomnia [29, 30]. Compared with traditional face-to-face CBT-I, it has advantages of convenience, flexibility, low cost, less dependence on doctors, and high patient compliance [31, 32]. Recent studies have shown that the effect of eCBT-I is equivalent to traditional face-to-face CBT-I [33]. English-speaking countries have provided several eCBT-I softwares (such as Sleepio and SHUTI), which show similar efficacy to standard CBT-I. However, there are few CBT-I suitable for Chinese people, and no relevant clinical research data has been found, so further exploration and verification are needed to test its efficacy [34–36].

In the early stage, we developed the insomnia TCM system for chronic insomnia based on WeChat public platform (software copyright, registration number: 2019SR0900086), which is used for TCM diagnosis, information collection, and management of chronic insomnia. It includes modules such as sleep self-test, personal files, tracking, and health education. Baduanjin exercise is a popular traditional Chinese fitness method that combines physical movement, breathing, and psychological adjustment. By stimulating the meridians and organs, [37, 38] it can improve sleep. Five-element music guided by the basic theory of traditional Chinese medicine has a unique curative effect in treating insomnia [39]. We can make a comprehensive analysis according to the syndrome differentiation of patients and choose the corresponding treatment strategy [40, 41].

In order to take advantage of the unique advantages of traditional Chinese medicine in treating insomnia and to explore suitable eCBT-I for Chinese people, this study realizes eCBT-I based on the health education module of the insomnia TCM system, incorporating the Baduanjin exercise and the five-element music, to form TCM-eCBT-I. This study aims to construct a convenient and feasible TCM-eCBT-I, to combine the advantages of acupuncture and TCM-eCBT-I, and to explore the chronic disease management model of acupuncture in the treatment of chronic insomnia. Meanwhile, the short-term and long-term efficacy of acupuncture, TCM-eCBT-I, and acupuncture combined with TCM-eCBT-I will be evaluated to provide information for clinical decision-making. Our hypothesis is that acupuncture combined with TCM-eCBT-I is more effective than TCM-eCBT-I or acupuncture single therapy.

## Methods and design

### Study design

This study is a single-center, open label, randomized controlled trial and will be completed in the insomnia clinic of the Acupuncture and Rehabilitation Department of the Affiliated Hospital of Nanjing University of Chinese Medicine from 01 June 2020 to 30 December 2021. This study follows the principles of Consolidated Standards of Reporting Trials (CONSORT), [42] and Standards for Reporting Interventions in Clinical Trials of Acupuncture(STRICTA) [43] will recruit 90 subjects and randomly assign them to the acupuncture group, the TCM-eCBT-I group, and the acupuncture combined with the TCM-eCBT-I group, at a 1: 1: 1 ratio. Subjects will be interviewed before recruitment, by completing a structured insomnia diagnosis process (refer to the 2017 China Sleep Diagnostic [44] - Insomnia Diagnosis Process) to diagnose chronic insomnia, and they will be screened for other sleep disorders, mental disorders, and serious physical diseases and through formal medical history and sleep symptoms. All subjects also need to take PSQI assessment and record one week sleep diary in order to obtain baseline assessment data. Subsequently, 4 weeks of treatment and 24 weeks of follow-up will be conducted.

**Fig. 1:**
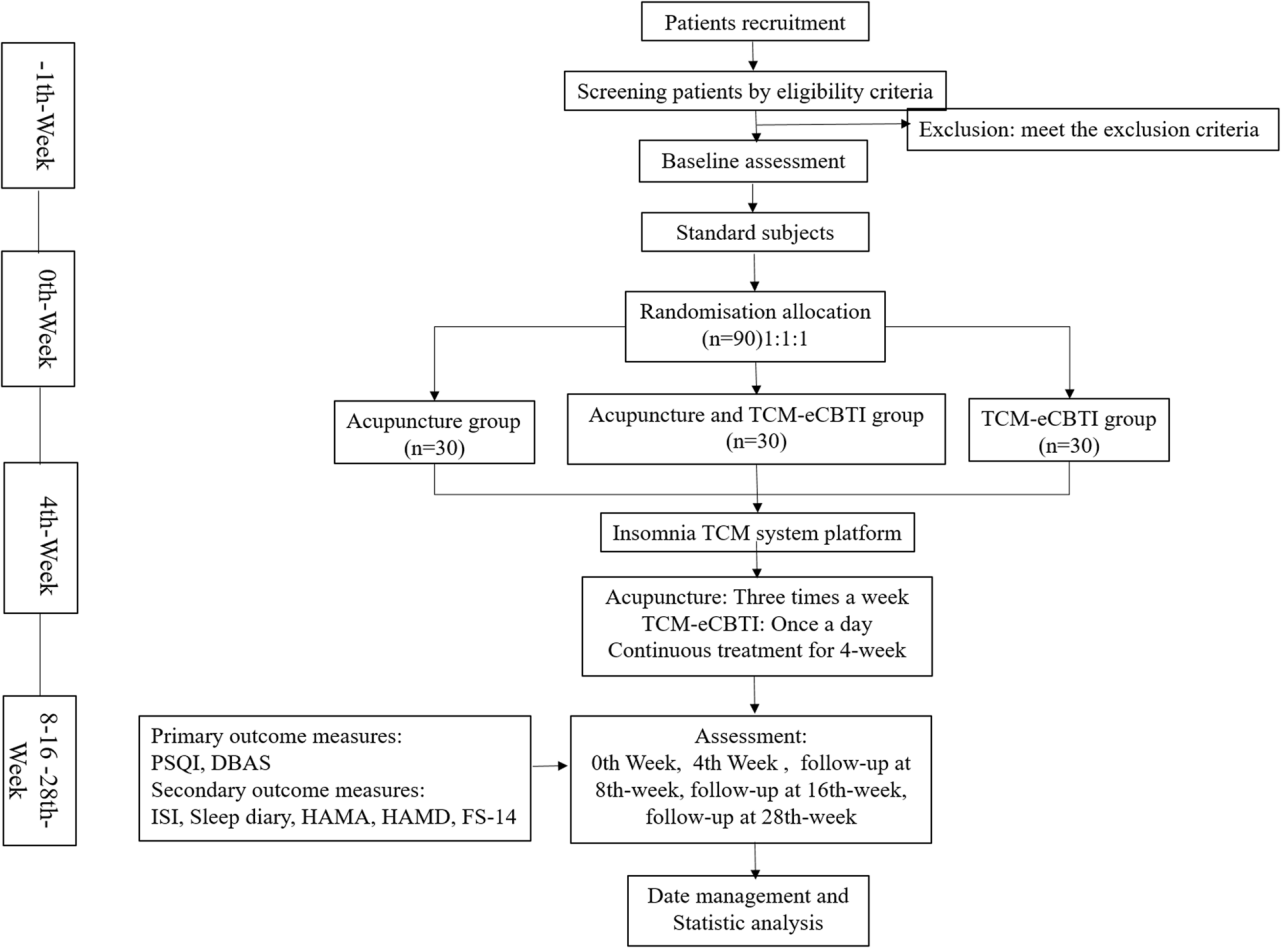
The test process

**Fig. 2:**
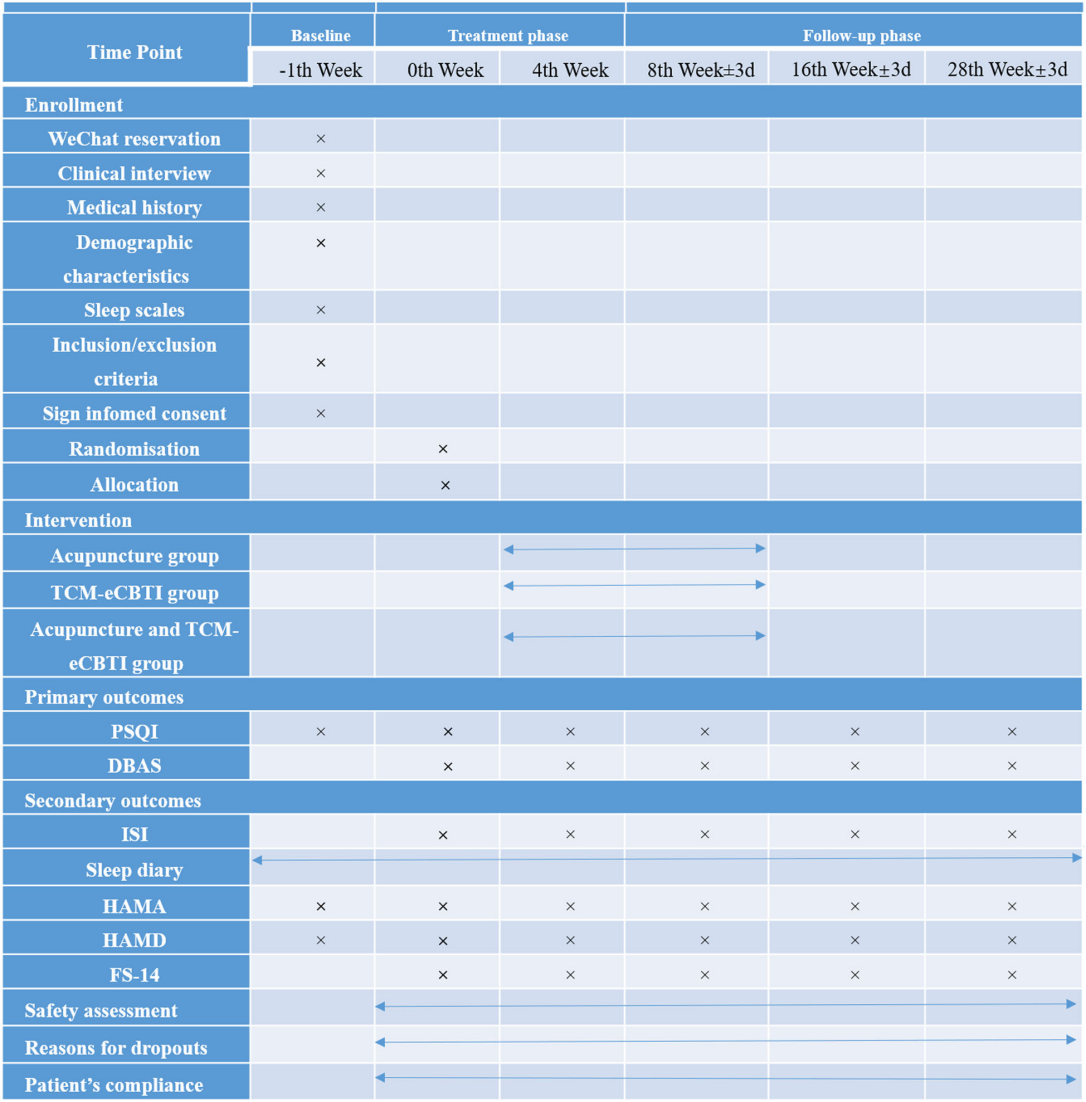
The time point of the assessment

All subjects will complete the following scale evaluation: PSQI, ISI, and sleep diary to evaluate sleep; DBAS to evaluate sleep cognition and belief; HAMA and HAMD to evaluate mental state; and FS-14 to evaluate mental and physical fatigue. The above scales will be evaluated online in the insomnia TCM system during the 0th week, 4th week, 8th week, 16th week, and 28th week. Subjects will sign an informed consent form after evaluating the conformity for inclusion. The test process is shown in Fig. 1, and the time point of the assessment is shown in Fig. 2.

### Study setting and recruitment

The recruitment will take place in the Affiliated Hospital of Nanjing University of Chinese Medicine, which is a large third-class grade A comprehensive hospital with good performance in clinical medicine and scientific research. Currently, it is an international acupuncture clinical training base of the World Health Organization and an international traditional medicine cooperation center. In this study, we will use the electronic screen recruitment and publicity of hospitals, tweets on insomnia TCM system, two-dimensional code scanning leaflets to fill in the registration form, and telephone registration to recruit subjects. The duration is 29 weeks for each subject, including 1 week of baseline period, 4 weeks of treatment period, and 24 weeks of follow-up.

### Randomization and allocation concealment

The statistical expert from the Faculty of Medical Statistics and Epidemiology from the Affiliated Hospital of Nanjing University of Chinese Medicine will use the statistical software SPSS (IBM SPSS statistics version 22.0, USA) to generate 3 different random number lists, each of which contains 30 numbers. The random numbers in each list will be sorted in ascending order. The randomization schedule calculated in no. 1–30 is the acupuncture group, no. 31–60 is TCM-eCBT-I group, and no. 61–90 is the acupuncture combined with TCM-eCBT-I group. According to the disclosed method, [45] the allocation sequences are enclosed in sealed envelopes with a unique identification number. The envelope will be sent to an investigator in charge of the group assignments. The investigator will specify in the envelope who and when to open the randomly assigned envelope. The number will appear on all report forms to maintain participant confidentiality. During the study, generation and distribution of random number lists, recruitment of subjects, practice by acupuncturists, assessment, data management, and statistical analysis will be independently conducted by different researchers.

### Blinding

This trial is an open-label trial. It cannot blind the patients and the acupuncturists. Participants are blinded to their group allocation but are informed that they had an equal chance of allocation to the three groups before study participation. The outcome assessor will also be blinded to the group allocation. Adverse reactions are carefully observed, and emergency unblinding is required if serious adverse reactions occur. Unblinding will be performed at the end of the trial to perform statistical analysis.

### Participants

#### Inclusion criteria

The inclusion criteria are as follows:

1. 18–60 years old, no gender limitation (either sex)
2. Meeting the diagnostic criteria for chronic insomnia in ICSD-3 [46]
3. PSQI score > 5 points [47]
4. Not accepted in the past month sedative and hypnotic drugs, acupuncture, and CBT-I treatment
5. Having smart terminals (such as smartphones, tablets, computers) and knowing how to use WeChat
6. No communication and cognitive impairment, voluntarily accept random grouping, and sign the informed consent

#### Exclusion criteria

Participants who fulfill any of the following criteria will be excluded:

1. Complicated with severe physical illness
2. Diagnosed with depression, schizophrenia, or other serious mental illness
3. Diagnosed with obstructive sleep apnea, restless leg syndrome, or other sleep disorders
4. In the past 2 weeks, there are irregular work schedules such as shift work or staying up late
5. Accompanied by fever, cough, and pain symptoms that seriously affect sleep
6. Alcohol or other drug abuse or dependence
7. Pregnancy or lactation which is not suitable to receive acupuncture treatment
8. Infectious diseases such as infectious hepatitis, tuberculosis, and AIDS
9. Participated in other clinical studies in the past 3 months

### Interventions

Subjects in the acupuncture group and the acupuncture combined with the TCM-eCBT-I group will all receive the same acupuncture treatment. Acupuncture will be conducted in the outpatient clinic of insomnia. The temperature in the clinic will be kept between 24 and 30°, and the environment is quiet and comfortable. The subjects will be told to relax, close their eyes, and have a rest.

#### Acupuncture group

**Fig. 3:**
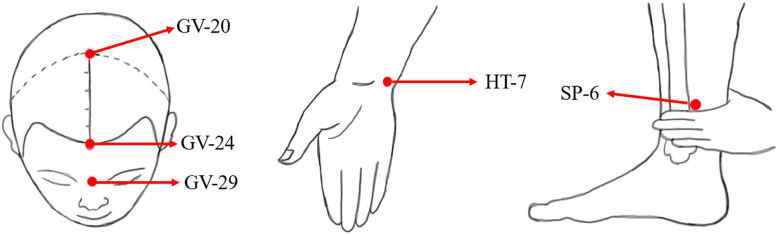
The location of acupoints

According to the theory of TCM, insomnia is closely related to mental disorders [48]. Based on the conclusion of previous systematic reviews [49, 50] and our clinical experience, [51] we will select Baihui (GV-20), Shenting (GV-24), Yintang (GV-29), Shenmen (HT-7, bilateral), and Sanyinjiao (SP-6, bilateral). The location of acupoints will be determined in strict accordance with the National Acupoint Standard of the People’s Republic of China in 2006 (GB/T 12346-2006) (Fig. 3) [52].

The subjects will be placed in the supine position, disposable sterile acupuncture needles with a length of 40 mm and a diameter of 0.30 mm will be selected (Huatuo, Suzhou, China), and the needles will be injected after disinfection. GV-20, GV-24, and GV-29 will be inserted 10 mm obliquely. HT-7 will be penetrated 10 mm straightly, while SP-6 will be perpendicularly punctured 15 mm. After piercing, thrusting and twirling will be used to achieve the sensation of “De qi.” After 30 min, take out the needle and squeeze it with a dry sterilized cotton ball. Subjects in the group which includes acupuncture will be treated 3 times a week for 4 weeks (Table 1).

**Table 1:** Details of acupuncture interventions

Intervention	Acupuncture treatment
**Points**	Baihui (GV-20), Shenting (GV-24), Yintang (GV-29), Shenmen (HT-7, bilateral), Sanyinjiao (SP-6, bilateral)
**Depth description**	GV-20, GV-24, and GV-29 being inserted 10 mm obliquely. HT-7 being penetrated 10 mm straightly, while SP-6 being perpendicularly punctured 15 mm
**Needle retention time**	30 min
**Needle type**	0.30 × 40 mm (Huatuo, Suzhou, China)
**Needle stimulation**	By thrusting and twirling to achieve the sensation of “De qi”
**Frequency and duration of treatment sessions**	Three times a week for 4 weeks

#### TCM-eCBT-I group

TCM-eCBT-I is based on sleep health education, cognitive therapy, and sleep restriction, incorporating the Baduanjin exercise and five-element music. Under the guidance of professional IT staff and clinical psychologists, the TCM-eCBT-I has been well developed and tested in previous research (Table 2).

1. Sleep health education, cognitive therapy, and sleep restriction: [53, 54] provide daily guidance for the subjects in the form of a sleep management manual.
2. Relaxation therapy of TCM: videos of Baduanjin exercise and five-element music will be sent to the subjects. Subjects are required to practice the Baduanjin exercise after 20:00 every evening, and listen to five-element music for half an hour before going to bed. This process will last for 4 weeks.

**Table 2:** Details of the TCM-eCBT-I interventions

Intervention	TCM-eCBTI	Therapy method
**Session 1: Sleep health education**	Standard behavioral guidelines for lifestyle and environmental factors related to insomnia: [1] avoid drinking excitable drinks, such as coffee and strong tea, in the afternoon [2]; avoid drinking alcohol before going to sleep [3]; avoid eating too much before going to sleep [4]; keep the bedroom quiet and dark [5]; keep the room temperature appropriate [6]; regular exercise.	Push instructions in the form of the sleep management manual at fixed time daily. Record treatment participation by daily clock-in.
**Session 2: Sleep cognition**	Reduce excessive expectations and demand for sleep, correct one’s own bad suggestion, dispel the idea of controlling the sleep process, and weaken the excessive self-attention.	
**Session 3: Sleep restriction**	Estimate the actual sleep time according to last week’s sleep status: sleep efficiency more than 85%, increase sleep time by 15 min next week. Sleep efficiency is 80.00~84.99%, keep the sleep time next week unchanged. Sleep efficiency less than 80%, reduce sleep time by 15 min next week.	
**Session 4: Baduanjin exercise**	Reference to the new standing Baduanjin exercise	Guide the patients to exercise the Baduanjin exercise at home through online text animation and teaching video on the TCM insomnia system. Record treatment participation by daily clock-in.
**Session 5: Five-element music**	The music is selected from the *Five Elements of Tian Yun* published by the Beijing Higher Education Audio-visual Press. According to the classification of the patient’s condition, select music from the five tunes of Gong, Shang, Jue, Zhi, and Yu.	Five-element music is played online through the TCM insomnia system before going to bed. Meditation relaxation training is performed while playing music. Record treatment participation by daily clock-in.

The above content is enhanced by various interactive functions, including personalized goal setting, feedback based on self-reports, interesting animations, and sleep education videos. In addition, when recording sleep diaries online, the insomnia TCM system will automatically remind users to record their performance, which could also be used to evaluate subjects’ compliance. Daily timekeeping will be used to increase their participation and to encourage them to follow the plan [55].

#### Acupuncture combined with the TCM-eCBT-I group

The subjects simultaneously receive acupuncture treatment (the same as the acupuncture group) and TCM-eCBT-I treatment (same as the TCM-eCBT-I group). Subjects will be treated 3 times a week for 4 weeks of acupuncture, and TCM-eCBT-I will be performed for 20 min every night for 4 weeks.

### Rescue medication

Throughout the whole trial, subjects will be told not to take any medicine or other treatment methods related to insomnia. If subjects suffer from insomnia all night, which seriously affects their work and life, they should take estazolam tablets (1 mg, Changzhou Four Drugs System, National Pharmaceutical Standard H32020699) temporarily under the guidance of researchers, allowing them to be used once a day for up to 3 days. The researcher will record detailed information (time, frequency, and dosage) of the drug use.

### Outcome measures

#### Primary outcome measures

The PSQI is a questionnaire to assess sleep quality. It consists of six aspects: sleep quality, sleep time, sleep efficiency, sleep disorders, hypnotic drugs, and daytime dysfunction. The total score of PSQI is 0–18 [56]. Sleep quality is negatively correlated with PSQI score. PSQI Chinese version has high reliability and validity and can be used as an effective tool for sleep screening and evaluation in clinical and scientific research [57, 58] PSQI > 5 points indicates that clinical treatment may be required [59] In this study, PSQI > 5 points will be used as the standard for chronic insomnia.

#### Secondary outcome measures

1. DBAS: Moran compiled the DBAS in 1993, which mainly includes five dimensions [60]: [1] beliefs about dysfunction of insomnia consequences, [2] belief that sleep is unpredictable and uncontrollable, [3] unrealistic sleep expectation, [4] misunderstanding of the causes of insomnia, and [5] misunderstanding of improving sleep style. A study shows that DBAS is not only used for qualitative assessment of sleep cognition, but also used as a tool to compare the efficacy before and after treatment [61] The Chinese version of the scale is also proved to have good reliability and validity [62].
2. ISI: The ISI is a self-filling scale developed by Morin and Espie (1993), [63] which is mainly used to evaluate insomnia in clinical research [64]. It is used to evaluate the subjective feelings of insomnia. Subjects fill out the form according to their sleep status of the past week. The range of scores is 0–28. The severity of insomnia is positively correlated with the ISI score [65]. Chinese version of ISI has been used to evaluate Chinese insomniacs and proved to have high reliability and validity [66].
3. Sleep diary: Subjects fill out a sleep diary for the night online after getting up in the morning, including the bedtime at night, the wake up time, the time to fall asleep, the number of times one wakes up after falling asleep, the feeling after waking up, the total sleep time, and the factors that interfere with sleep. Subjects can record their own sleep behavior patterns and daytime conditions affected by sleep through a sleep diary. Through the analysis by clinicians, they can intuitively grasp the sleep information of patients, which is of great significance for the diagnosis and evaluation of insomnia [67].
4. HAMA: The HAMA was formulated by Hamilton in 1959 [68]. This test uses a 14-item version and 5-level scoring of 0–4 points and scores from both physical and mental aspects. The severity of anxiety symptoms is positively correlated with the HAMA score. Subjects with a clinical score of more than 21 are regarded as definitely anxious [69].
5. HAMD: The HAMD was put forward by Hamilton in 1960. This study uses a 17-item version containing 7 factors, including despair, anxiety, somatization symptoms, and sleep disorders. The severity of depression is positively correlated with the HAMD score. More than 24 points indicate depression [70].
6. FS-14: The FS-14 was proposed by Trudy Calder and other scholars in 1992. It consists of 14 items. The two dimensions of physical fatigue and mental fatigue are projects 1–8 and projects 9–14. The physical fatigue score is 8 points; the mental fatigue score is 6 points. The severity of fatigue is positively correlated with the FS-14 score [71].

### Safety assessments

Throughout the whole study, we will evaluate the safety through the table of adverse events [72]. The table should record the following elements for adverse events: time point, severity, measures taken, relatedness to treatment, time to normal, and time to symptoms and signs disappear. The adverse reactions of acupuncture mainly include local hematoma, dizziness, and blood stasis. If symptoms are seriously worsening, the treatment will terminate. No adverse effect of eCBT-I has been reported.

### Sample size

We adopted the PASS software method: this study was a randomized controlled trial, and the three groups were the acupuncture group, combined group, and CBTI group. The PSQI reduction rate of the subjects was the observed outcome index. According to the results of our preliminary test, it was expected that the effective rate of the acupuncture combined with TCM-eCBT-I group was 75%. The treatment rate of the acupuncture group was 65% and that of the TCM-eCBT-I group was 45%. The degree of certainty is 90%. The PASS15 software was used to calculate the total sample size of the three groups, *N* = 72 cases. Considering 20% of the cases of lost follow-up and rejection, a total of 90 cases were needed for the three groups, among which 30 cases were needed for each group.

### Statistical analysis

The statistics and analysis of all data will be conducted by an independent researcher who does not participate in the experiment. We will use the SPSS 22.0 statistical software for data analysis. Descriptive statistics will be used to describe the demographic data of sample groups, while the normality of the distribution of each parameter will be checked by histogram and the Kolmogorov-Smirnov test. A chi-squared test will be used to assess the sample characteristics and gender.

The measurement data were expressed as mean ± standard deviation (SD) or median (interquartile range) [M (IQR)]. The analysis of variance will be used to test the between-group differences for the PSQI, ISI, DBAS, HAMA, HAMD, FS-14 scores, and sleep diary. As to the comparisons of the three groups, normality and homogeneity of variance are analyzed by variance analysis. If the variance is not uniform or normality is not conformed, the Mann-Whitney *U*-test will be used. Counting data is chi-square test. All statistical tests will be conducted bilaterally, and *P* < 0.05 indicates statistical significance. Data analysis will follow the intention-to-treat principle including participants who are randomly assigned. Missing data will be input with the last observation carried forward method.

### Data collection and management

Data will be collected and managed during the baseline period, before and after treatment, and during the follow-up period. Each assessment includes [1] a 20-min sleep-related online scale assessment in the insomnia Chinese medicine system, completed independently by subjects, and [2] a sleep diary prompted by the insomnia platform to subjects at 8:00 a.m.

### Privacy protection for participants

Only researchers and supervisors in this study may access the personal medical records of the subjects, and they will sign a “researcher statement” or “confidentiality commitment.” Data processing will use the method of “data anonymity,” omitting the information that can identify the personal identity of the subjects. If any subjects want to quit, the researchers will ask if they are willing to complete the final evaluation and record the last treatment time.

### Quality control

All researchers will receive special trainings; introduction of clinical consultation, acupuncture operation, and insomnia TCM system; and instructions on how to fill in the case report form. The acupuncturists hold a master’s degree in acupuncture and massage from the Nanjing University of Chinese Medicine. The assessors will receive professional training from psychologists. The data analysts will receive training in data input, coding, security, and storage. Statisticians will be trained in data evaluation and analysis. This study will be supervised by the Scientific Research Department of the Affiliated Hospital of Nanjing University of Chinese Medicine, so as to protect the rights and interests of subjects, to ensure the truthfulness, accuracy, and completeness of research data records and reports and to ensure that this study complies with the approved agreements and relevant regulations. The causes of loss will be recorded in detail by testing.

### Ethics and dissemination

This study is conducted in accordance with the Helsinki Declaration and has been approved by the Institutional Review Board of the Affiliated Hospital of Nanjing University of Chinese Medicine (2020 NL-018-02). All subjects and their families will be fully informed of the test situation and decide whether to participate in it. If the subjects agree to participate, they will be required to sign an informed consent. Any revision of this agreement will be reported and approved by the Institutional Review Board of the Affiliated Hospital of Nanjing University of Chinese Medicine. The results will be disseminated by publishing in an influential medical journal with an online version. We also plan to share the results with medical professionals, the public, and relevant organizations in the form of a conference report after it is published.

## Discussion

The reason why insomnia is easy to become chronic is related to the patients’ bad beliefs and attitudes toward sleep, such as repeatedly worrying about the adverse consequences of insomnia and unrealistic expectations of sleep. These bad cognitions will lead to the patients’ nervousness and anxiety, leading to persistent over-awakening and aggravating insomnia. On the other hand, bad sleep habits, such as irregular sleep and excessive bedtime during the day, can also lead to circadian rhythm disorder, leading to and maintaining insomnia. CBT-I can improve insomnia by reducing poor cognition and control of sleep. It is suggested to be the first choice in treating insomnia [73]. However, due to the complicated process, long treatment cycle, high medical expenses, and the shortage of professional therapists in China, many insomniacs cannot benefit from it. In addition, domestic scholars have studied that after 4 weeks of eCBT-I treatment, only 57.9% of patients completed the treatment, suggesting that compliance for eCBT-I is low [54].

Acupuncture is effective, safe, and convenient, with high compliance of the patients in treating insomnia. However, because the patients’ bad sleep behavior and wrong sleep cognition have not been effectively corrected, the effect of acupuncture is only remarkable in the short term, but unstable in the long term. In addition, long-term acupuncture treatment is rarely carried out in most clinical studies, and the long-term effect of acupuncture on insomnia is unclear. Acupuncture and CBT-I have different key characteristics and behavioral requirements, which may be different in improving sleep, and each has its own advantages [74]. Combining the advantages of acupuncture and eCBT-I, this study actively corrects the unreasonable cognition of patients on sleep, develops good sleep hygiene habits, and achieves long-term and stable curative effects. Long-term follow-up will be conducted to provide clinicians with the best plan for insomniacs.

A comparative study of acupuncture and CBT-I treatment of insomniacs with cancers shows that both treatments have shown good results in improving sleep through different mechanisms [75]. Previous studies have found that auricular acupuncture has a greater impact on sleep duration of insomniacs than CBT-I, [76, 77] while other studies have also shown that CBT-I was superior to acupuncture in treating insomnia, and showed similar improvements in fatigue, mood, and quality of life [78]. Due to the small number of studies, it is not yet possible to draw reliable conclusions.

In this study, the insomnia TCM system is integrated into the traditional exercises of Baduanjin exercise and five-element music to form TCM-eCBT-I, which are more suitable for Chinese people. Second, the effects of acupuncture, TCM-eCBT-I, and acupuncture combined with TCM-eCBT-I on the subjects’ subjective sleep, sleep attitude and belief, anxiety, depression, and daytime dysfunction will help doctors choose more appropriate treatments for insomniacs by evaluating the sleep-related scale and sleep diary. At the same time, the application of the insomnia TCM system provides a reference for exploring the chronic disease management mode of insomnia. According to the guidelines, the first 6 months after stopping treatment is a high-risk period for insomnia recurrence [79]. Therefore, long-term follow-up will be conducted through an online sleep diary and sleep scale score of the insomnia TCM system to observe its long-term curative effect. Of course, there are some localization and regrettable in the study. Recruitment of this study is limited to one center of Chinese First-class hospitals, and the sample size is too small so the results may not be applicable to other levels of hospitals or other countries. This study cannot blind subjects, which may lead to bias and affect the results. These limitations should be improved and supplemented in future studies.

## Trial status

This trial is recruiting patients now. Participant recruitment started on 20 October 2020 and is expected to end on 31 December 2021. This trial was registered in the Chinese Clinical Trial Registry on 17 May 2020. The registration number is ChiCTR2000032960. Protocol version 2, 31 March 2020.
